# Supplementary figures and images for: Prostate-specific membrane antigen in circulating tumor cells is a new poor prognostic marker for castration-resistant prostate cancer
Source: PLoS One. 2020 Jan 27;15(1):e0226219. doi: 10.1371/journal.pone.0226219 (PMC6984691; doi:10.1371/journal.pone.0226219)

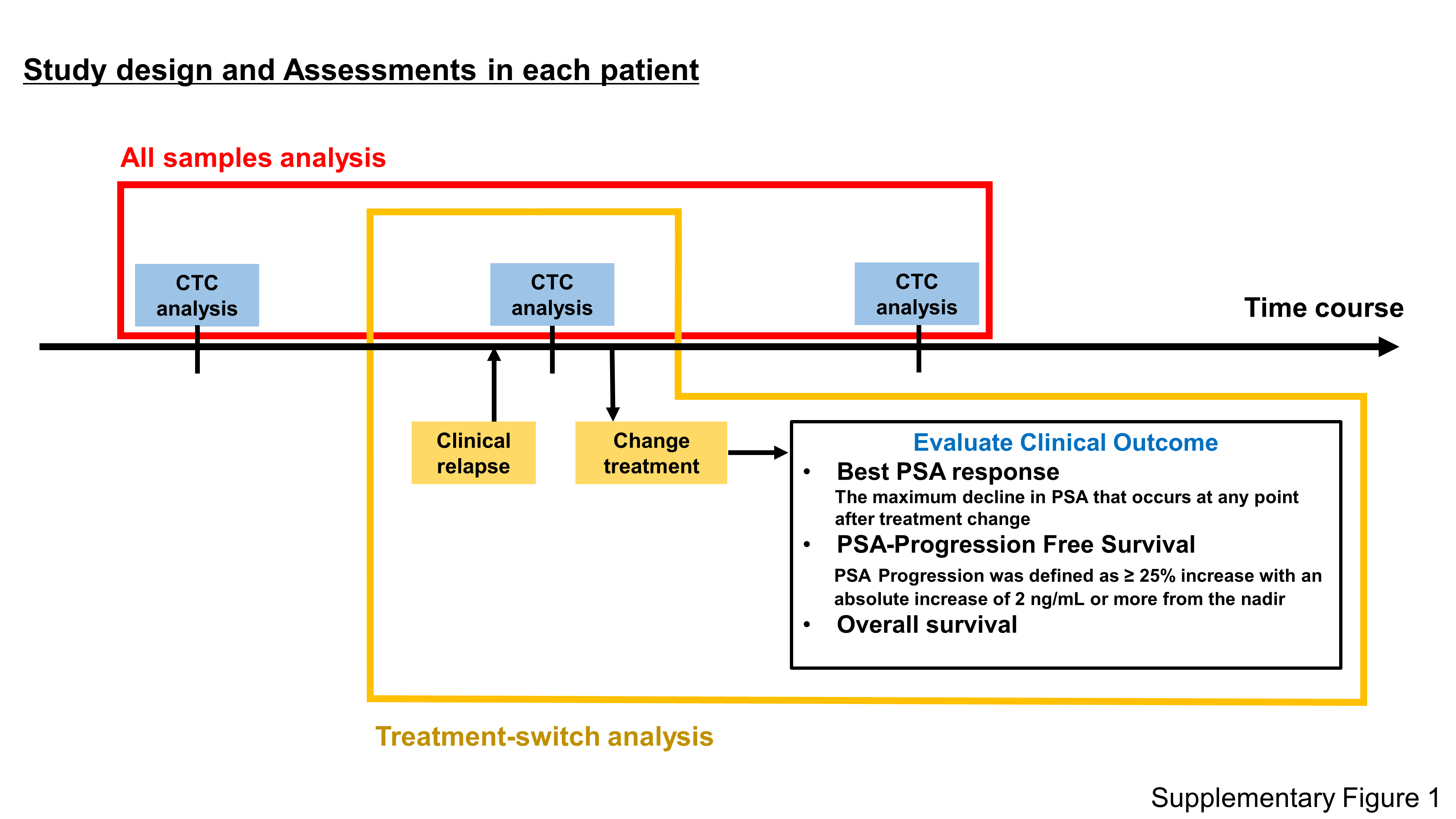

Supplement: S1 Fig — (TIF) [file pone.0226219.s001.TIF]
